# Low levels of exosomal-miRNAs in maternal blood are associated with early pregnancy loss in cloned cattle

**DOI:** 10.1038/s41598-017-14616-1

**Published:** 2017-10-30

**Authors:** T. H. C. De Bem, J. C da Silveira, R. V. Sampaio, J. R. Sangalli, M. L. F. Oliveira, R. M. Ferreira, L. A. Silva, F. Perecin, W. A. King, F. V. Meirelles, E. S. Ramos

**Affiliations:** 1 Department of Genetics, Ribeirao Preto Medical School, University of Sao Paulo, Ribeirao Preto, Brazil; 2 Department of Veterinary Medicine, Faculty of Animal Science and Food Engineering, University of Sao Paulo, Pirassununga, Sao Paulo Brazil; 3 Department of Animal Reproduction, Faculty of Veterinary Medicine and Animal Science of University of Sao Paulo, Sao Paulo, Brazil; 4 Department of Biomedical Science, Ontario Veterinary College, University of Guelph, Ontario, Canada

## Abstract

Nuclear reprogramming mediated by somatic cell nuclear transfer (SCNT) has many applications in medicine. However, animal clones show increased rates of abortion and reduced neonatal viability. Herein, we used exosomal-miRNA profiles as a non-invasive biomarker to identify pathological pregnancies. MiRNAs play important roles in cellular proliferation and differentiation during early mammalian development. Thus, the aim of this study was to identify exosomal-miRNAs in maternal blood at 21 days of gestation that could be used for diagnosis and prognosis during early clone pregnancies in cattle. Out of 40 bovine-specific miRNAs, 27 (67.5%) were with low abundance in the C-EPL (Clone - Early pregnancy loss) group compared with the C-LTP (Clone - Late pregnancy) and AI-LTP (Artificial Insemination - Late pregnancy) groups, which had similar miRNAs levels. Bioinformatics analysis of the predicted target genes demonstrated signaling pathways and functional annotation clusters associated with critical biological processes including cell proliferation, differentiation, apoptosis, angiogenesis and embryonic development. In conclusion, our results demonstrate decreased exosomal-miRNAs in maternal blood at 21 days of gestation in cloned cattle pregnancies that failed to reach term. Furthermore, the predicted target genes regulated by these 27 miRNAs are strongly associated with pregnancy establishment and in utero embryonic development.

## Introduction

Generation of animal clones by somatic cell nuclear transfer (SCNT) is a technology with potential applications in both agriculture and medicine^1^. Specifically, in medicine, animals expressing human-specific proteins have been successfully produced using SCNT and transfected donor cells^2,3^, and the use of this technology has improved the efficiency of transgenic animal generation when compared with pronuclear microinjection^4,5^.

However, several research groups have reported difficulties in obtaining viable SCNT-derived animals^6–8^. Bovine embryos generated by SCNT generally show increased rates of abortion and reduced neonatal viability^9,10^. Most embryonic loss occurs during the implantation period (first trimester), often due to problems in placenta vascularization and cotyledon malformation^11^. Placental dysfunction has been extensively reported in clone pregnancies^7,11^, and there is a high correlation between perinatal mortality and abnormal placental function in SCNT calves^12^. In addition to placental functionality, pregnancy success depends on exact timing of the maternal-fetal interactions as well as a perfectly orchestrated transcriptional pattern within the placenta^12^.

Interestingly, placental transcription profiles revealed many microRNAs (miRNAs) that are unique from the placenta^13,14^. MiRNAs have tissue-specific abundance levels, and therefore they are excellent candidates for use as non-invasive biomarkers^15,16^. MiRNAs are small non-coding RNA molecules that are approximately 22 nucleotides in length and modulate gene expression by binding the 3′UTR mRNA region and degrading or repressing target mRNAs^17^. MiRNAs play important roles during cellular proliferation and differentiation, apoptosis, and disease progression during early mammalian development^18^. In addition to the miRNA roles within the cellular cytoplasm, recent studies involving free-circulating miRNAs^19^ or miRNAs bound within extracellular vesicles membranes known as exosomes^20,21^, offer possibilities to develop molecular biomarkers for pregnancy disorders.

Exosomes are small vesicles approximately 40–160 nm in diameter that are formed from intraluminal vesicles inside late endosomes or multi-vesicular bodies^22^. The molecular composition of an exosome reflects its origin and may include proteins, mRNAs and/or miRNAs^23^. Exosomes are considered as long-distance signal transporters mediating cell-to-cell communication. Thus, the occurrence of pregnancy-associated diseases has been linked with altered exosomal-miRNA profiles. However, the functions of most of these circulating-miRNAs remain unknown^13,14,19^.

In cattle, some studies have evaluated free-circulating or exosomal-miRNAs in the blood of embryo-recipient cows and their associations with early pregnancy development or, more specifically, with early pregnancy loss^24^. A recent study showed that the levels of some miRNAs, including bta-miR-496 and bta-miR-125a, vary considerably during the pre-implantation embryo development, suggesting that the transition from maternal-to-zygote transcription may change the levels of circulating miRNAs^25^. Additionally, other miRNAs, such as bta-miR-27a and bta-miR-92b, have variable abundance levels during placental development and are associated with trophoblastic differentiation and vascularization^26,27^. A profile of miRNA levels from the plasma of artificially inseminated pregnant dairy cows during the early stages of pregnancy versus non-pregnant cows identified changes in specific miRNAs. The levels of bta-miR-26a increased from gestational days 16 to 24 and, according to the authors, these changes may reflect a disturbance in the miRNA abundance patterns in one or more body tissues and might play an important role in pregnancy outcomes^24^.

Based on these findings, our study sought to profile exosomal-miRNAs in the blood of pregnant cattle that had received SCNT- or *in vivo*-produced embryos. The exosomal-miRNAs were profiled on the 21^st^ day of gestation (D21) and correlated with pregnancy outcome. Our hypothesis is that cows gestating poor-quality embryos possess different levels of miRNAs compared with cows that maintain the gestation until term. This is the first study to evaluate exosomal-miRNAs in the blood of SCNT embryo-recipient cows during the early stages of pregnancy. Thus, this study can contribute to an improved understanding of developmental failures during pregnancy.

## Results

### *In vitro* and post implantation developmental of blastocysts produced by SCNT

For miRNA analysis in the peripheral blood of pregnant recipients on the 21^st^ day of gestation, three SCNT runs were conducted. A total of 733 oocytes were matured *in vitro*, and the 1^st^ polar body (PB) extrusion rate was 66.6% (n = 488). The fusion and blastocyst rates on the 7^th^ day of culture (D7) were 82.0% (n = 282) and 32.3% (n = 91), respectively. The pregnancy rate (n = 11; 24.0%) was observed on the 28^th^ day of gestation (Table 1).

**Table 1:** Pre-implantation development of SCNT blastocysts.

Replicates	Oocytes	1^st^ PB		Reconstructed	Fusion		Blastocysts	
	N	N	%	N	N	%	N	%
1	223	146	65.5	113	102	90.3	42	41.2
2	238	155	65.1	106	89	84.0	26	29.2
3	272	187	68.7	125	91	72.8	23	25.3
Total	733	488	66.6	344	282	82.0	91	32.3

The percentages of cleaved embryos and blastocysts were estimated in relation to the number of successfully fused oocytes. The total values are representative of three experimental replicates. (PB) polar body.

### Pregnancy monitoring

**Figure 1:**
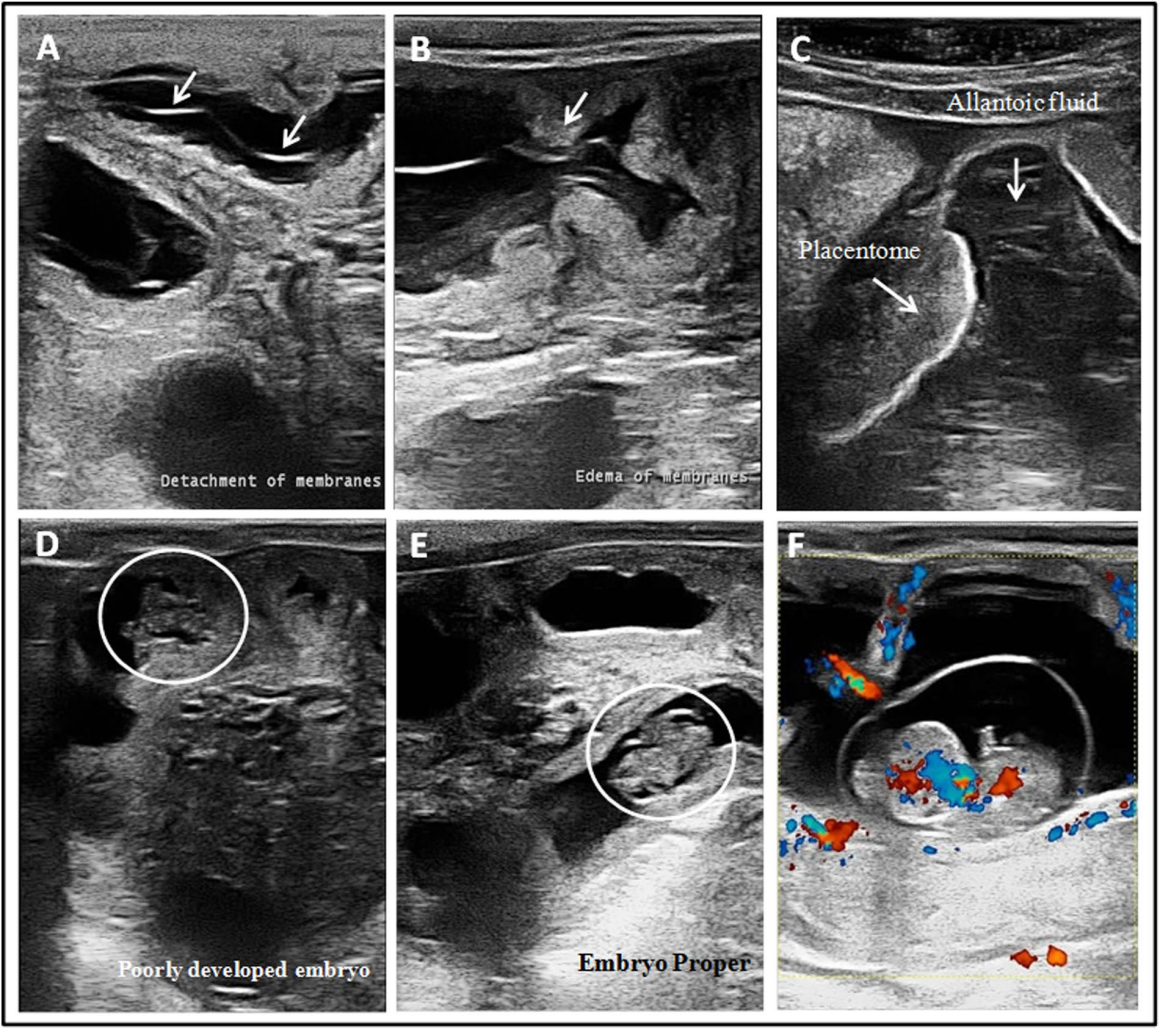
Images obtained by ultrasound during clone pregnancy monitoring. (**A**) The arrows indicate detachment of the membranes at day 55 of gestation. (**B**) Arrows indicate edema of fetal membranes at day 55 of gestation. (**C**) Arrows indicate placentome and allantoic and hyperechoic fluid at day 71 of gestation. (**D**) Circle indicates a poorly developed embryo at day 30 of gestation. (**E**) Circle indicates a proper embryo at day 34 of gestation. (**F**) Normal vascularization in a placenta and in a viable fetus visualized at day 47 of gestation by color Doppler. Images (**A**–**D**) are from gestational problems found in the C-EPL group. Images (**E** and **F**) are from embryos of the C-LTP group where no problems were encountered. Note the normal vascularization in the placenta and fetus.

A single SCNT blastocyst was transferred into each recipient cow, and pregnancy establishment was evaluated during the early stages of embryo development by ultrasound visualization of the fetal heartbeat on the 28^th^ day of gestation (D28). The pregnancies were allowed to develop to term or until spontaneous abortion and were grouped according to the pregnancy outcome. The average days to detect the embryonic vesicle for the C-EPL and C-LTP groups were 26.3 and 27.3 days, respectively. Heartbeat detection and the average heart rate for the C-EPL and C-LTP groups were 27 days and 177 bpm and 28.6 days and 164 bpm, respectively. Two embryos were poorly developed in the C-EPL group, whereas only one embryo had apparently normal development. Additionally, the pregnancies were spontaneously aborted on days 32, 48 and 86 of gestation. In contrast, all embryos analyzed from the C-LTP group had apparently normal development, according to ultrasound analysis, and reached to term. We did not observe differences in placental vascularization between the C-EPL and C-LTP groups, and only pregnancies from the C-EPL group showed placental edema, detachment of fetal membranes, and hyperechoic allantoic and amniotic fluids. All of the results above are summarized in Table 2 and demonstrated in Fig. 1.

**Table 2:** Characteristics of cloned pregnancies obtained using ultrasound image analysis.

	Clone - Early pregnancy loss	Clone - Late pregnancy
Embryonic vesicle detection	26.3 d	27.3 d
Heartbeat detection	27 d (177 bpm)	28.6d (164 bpm)
Conceptus development	Normal (1) and poorly developed (2)	Normal (3)
Vascularization	Normal (3)	Normal (3)
Other Findings	Edema and Detachment of membranes, hyperechoic allantoic and amniotic fluids (2)	N/D

Three gestations for each group were monitored by ultrasound examination. The embryonic vesicle analysis and heartbeat detection are represented as a mean number of days. (N/D) no problems were found.

### Exosomal-miRNA abundance levels in maternal plasma

**Figure 2:**
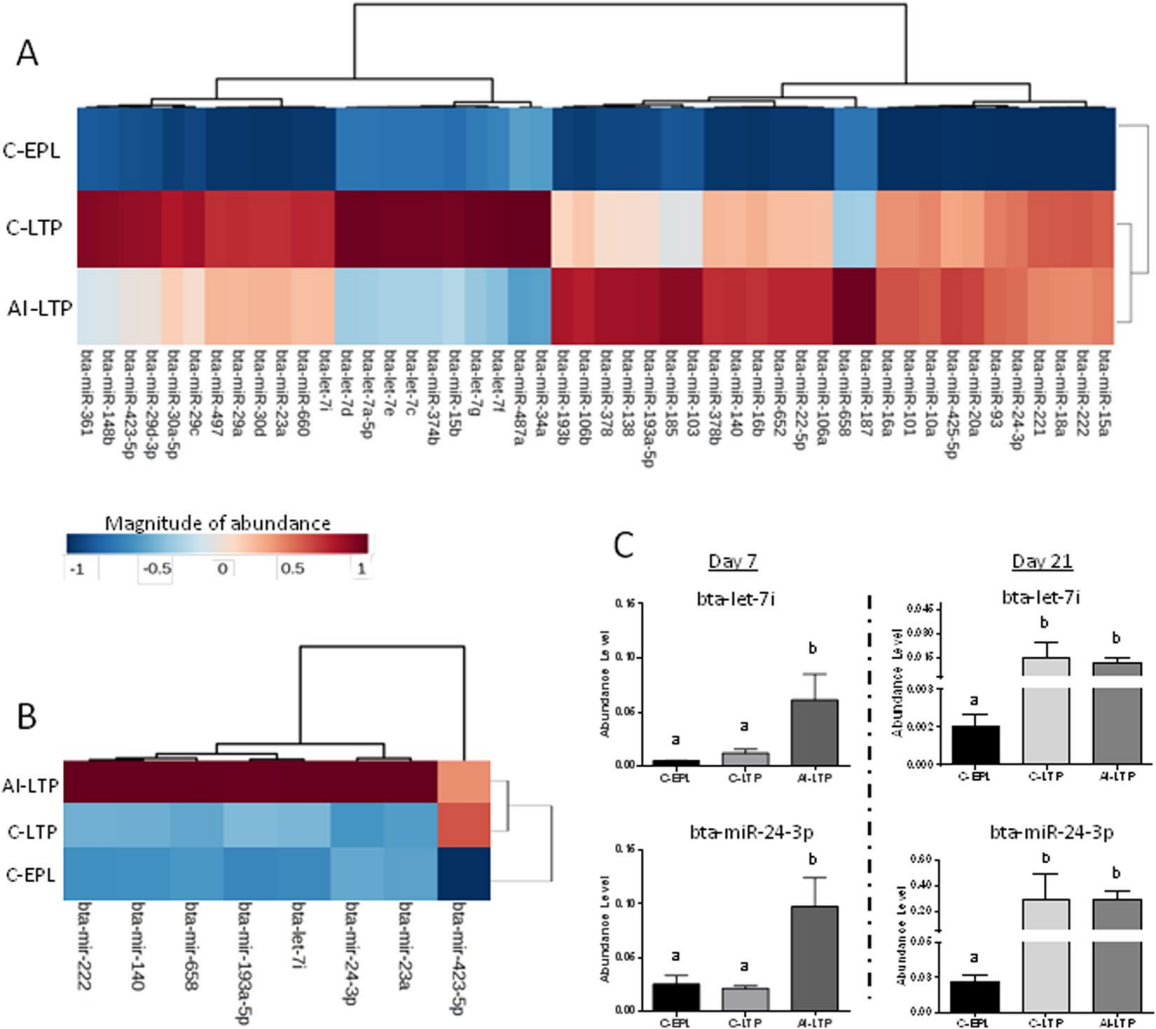
(**A**) Heatmap depicting the average Ct values from the 48 miRNAs with different abundance levels in the maternal blood at the 21^st^ day of gestation among the following three groups evaluated: Clone - Early Pregnancy Loss (C-EPL), Clone - Late Pregnancy (C-LTP), Artificial Insemination - Late Pregnancy (AI-LTP). (**B**) Heatmap presenting the average Ct values of eight miRNAs with different abundance levels in the maternal blood on the 7^th^ day among the same three groups. The higher miRNA levels are shown in red, whereas the lower miRNA levels are in blue. (**C**) Abundance levels of two miRNAs (bta-let-7i and bta-miR-24-3p) on the 7^th^ and the 21^st^ days of gestation among the three groups evaluated.

We quantitatively profiled the exosomal-miRNAs and detected a total of 275 miRNAs in the maternal blood on the 21^st^ day of gestation in the three groups evaluated (C-EPL, C-LTP and AI-LTP). Statistical analysis showed that 48 (17.4%) miRNAs were observed to have different abundance levels among the three experimental groups with their abundance levels represented in the heatmap (Fig. 2A). To verify whether the different abundance levels of these 48 exosomal-miRNAs were due to the animals themselves or to the establishment of pregnancy, we quantitatively profiled these 48 miRNAs from recipient cow maternal blood samples collected at the time of embryo transfer (D7). We detected that eight exosomal-miRNAs (bta-let-7i, bta-miR-140, bta-miR-193a-5p, bta-miR-222, bta-miR-23a, bta-miR-24-3p, bta-miR-423-5p and bta-miR-658) were with different abundance levels among the cows at embryo transfer. The abundance levels are represented in the heatmap (Fig. 2B). Among these eight miRNAs, the levels of two miRNAs (bta-let-7i and bta-miR-24-3p) changed during the course of pregnancy from days-7 to 21 in the C-LTP group only, as shown in the graphics below (Fig. 2C). Thereafter, we removed these eight miRNAs, and only the remaining 40 miRNAs with different abundance levels on the 21^st^ day of gestation were considered as potentially related to the pregnancy outcome. Interestingly, from these 40 miRNAs with differential abundance levels on the 21^st^ day of gestation, 27 miRNAs (67.5%) had similar highly abundance levels in the C-LTP and AI-LTP groups and low abundance level in the C-EPL group. The relative levels are represented in the heatmap (Supplementary Figure 1). Additionally, there were no miRNAs with high abundance levels in the C-EPL group compared with the two other groups (C-LTP and AI-LTP).

### Group distribution by principal component analysis (PCA)

**Figure 3:**
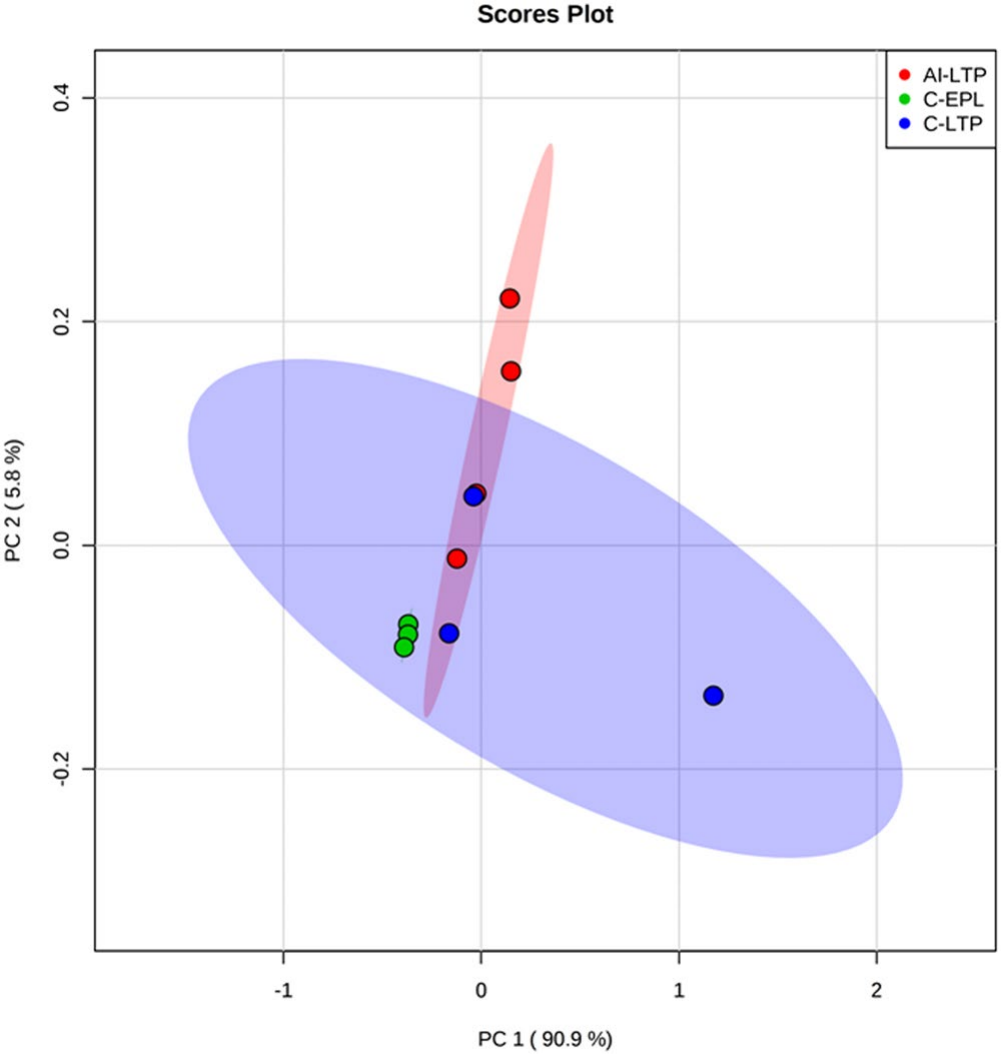
Principal component analysis (PCA) plot using the data from the 27 exosomal-miRNAs with different abundance levels on the 21^st^ day of gestation among the three groups analyzed by qRT-PCR. C-EPL: green dots; C-LTP: blue dots; and AI-LTP: red dots.

We carried out PCA of the 27 exosomal-miRNAs with low abundance in the C-EPL group in relation to other groups, C-LTP and AI-LTP, which both had high abundance levels of these exosomal-miRNAs. Figure 3 illustrates the individual distribution of each sample analyzed from each group after the PCA application. The principal component 1 (PC 1) had a value of 90.9%, whereas principal component 2 (PC 2) had a value of 5.8%. Both components combined had a sum of 96.7%. A clear pattern emerged among the samples from the C-EPL group, which were clustered together and presented a homogeneous distribution pattern. In contrast, PCA analysis of the C-LTP and AI-LTP groups did not result in a well-defined distribution, and these groups appeared to have heterogeneous distributions. Additionally, a similar analysis of 40 miRNAs with different abundance levels among the C-EPL, C-LTP and AI-LTP groups was performed (Supplementary Figure 2).

### Bioinformatics analysis of miRNA target genes

**Figure 4:**
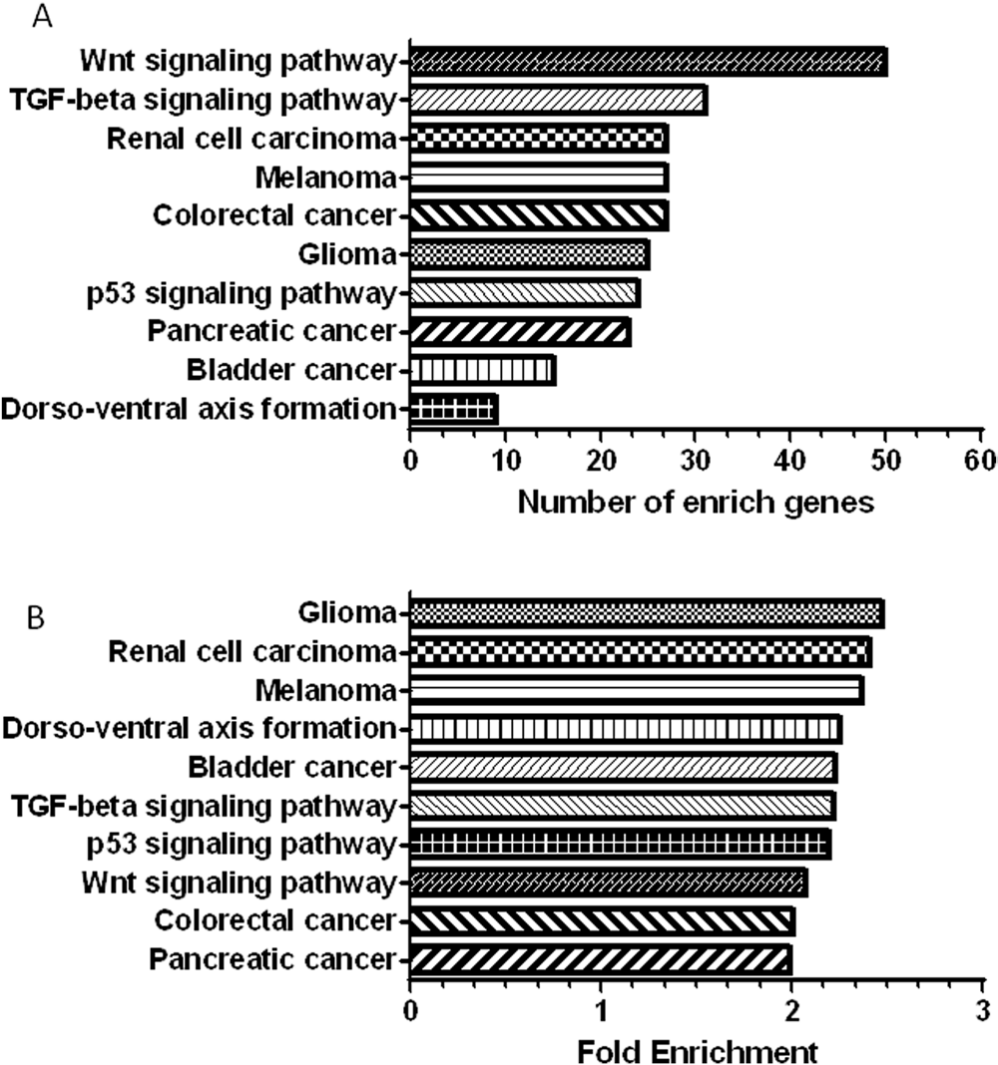
The top 10 signaling pathways involved in the 27 miRNAs with different abundance levels among the groups C-EPL (low levels), C-LTP (high levels) and AI-LTP (high levels). (*A*) The signaling pathways (P < 0.05) are arranged according to the number of involved genes. (*B*) The signaling pathways are arranged according to gene fold enrichment. The fold enrichment score reflects the involvement of the genes in each signaling pathway.

**Figure 5:**
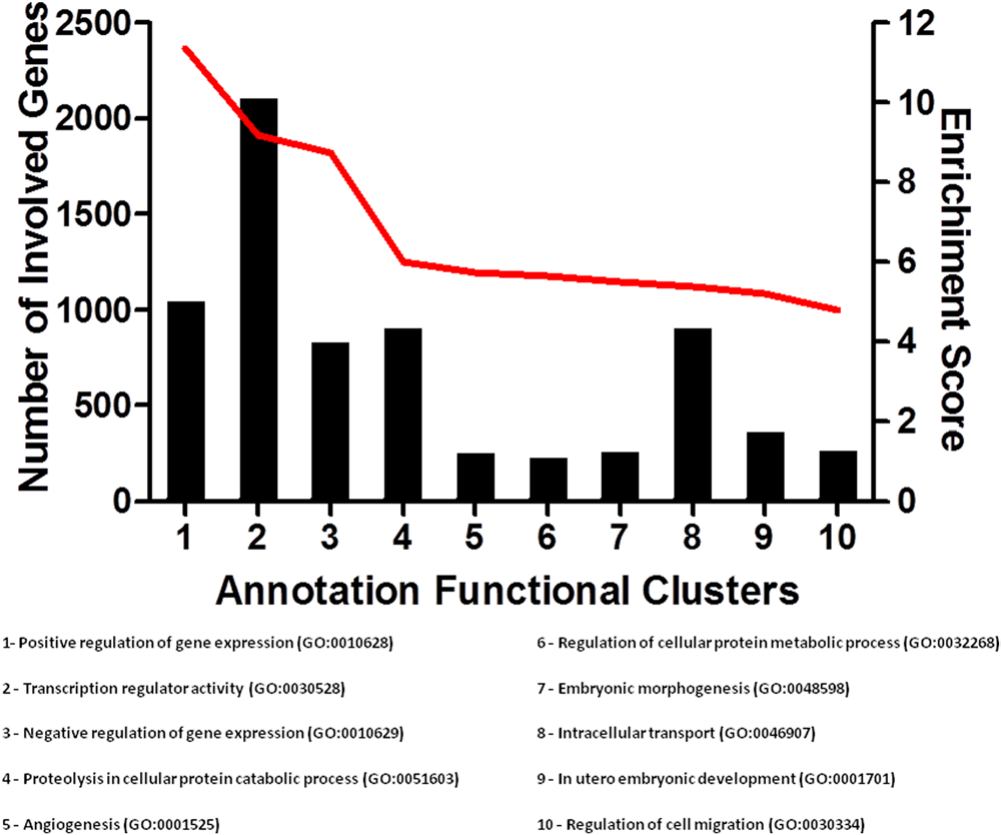
The graphic represents the top 10 functional annotation clusters from a total of 655 clusters identified. The black bars indicate the numbers of genes involved (axis Y left) in each cluster, and the red line indicates the enrichment score (axis Y right). The gene ontology reference (GO) is represented at the end of each cluster.

We performed a functional analysis of the predicted target genes from the 27 miRNAs that had low abundance levels in the C-EPL group compared with the C-LTP and AI-LTP groups, which both had high abundance levels of these miRNAs. The functional analysis was intended to identify possible signaling pathways associated with the miRNAs of interest. We also sought to determine functional annotation clusters related to the miRNAs. The functional annotation was performed using transcript accession numbers (Ensembl Transcript ID), and 2995 genes were analyzed. A total of 44 signaling pathways were found (Supplementary Table 1), and we selected the top 10 signaling pathways for analysis (Fig. 4A and B). The target genes identified in cellular or disease signaling pathways were as follows: 50 genes in Wnt signaling pathway; 31 genes in TGF-beta signaling pathway; 27 genes in renal cell carcinoma; 27 genes in melanoma; 27 genes in colorectal cancer; 25 genes in glioma; 24 genes in p53 signaling pathway; 23 genes in pancreatic cancer; 15 genes in bladder cancer; and nine genes in the dorso-ventral axis formation (Fig. 4A). For the functional analysis, 655 clusters involved with the miRNA target genes were identified (Supplementary Table 2), and we also selected the top 10 functional annotation clusters (Fig. 5). All of these signaling pathways and their functional clusters were statistically significant (P < 0.05). The fold enrichment values for all of these signaling pathways and the enrichment scores of all functional annotation clusters are represented in Fig. 3B and Fig. 4, respectively.

## Discussion

We screened exosomal-miRNA payloads from the plasma of pregnant recipients carrying a SCNT embryo on the 21^st^ day of gestation in comparison with a group of cows impregnated by AI. This approach allowed us to identify some signaling pathways relevant to pregnancy establishment and maintenance in cattle. We detected 275 miRNAs among the C-EPL, C-LTP, and AI-LTP experimental groups. Among these miRNAs, 40 were observed to have different abundance levels among groups on day 21 but not day 7. Curiously, the exosomal-miRNA abundance levels were similarly high between the C-LTP and AI-LTP groups but low in C-EPL recipients that experienced early pregnancy abortion (first trimester). The data presented here demonstrate that exosomal-miRNA content is highly correlated with the potential for a pregnancy to reach term in cattle.

MiRNAs play key roles in the regulation of genes among important signaling pathways. In humans, different studies have identified circulating miRNAs associated with pregnancy problems^13,14^. However, in cattle, the literature is quite sparse, and only a few studies have assessed circulating miRNAs in maternal blood from embryo transfer recipients, and even fewer have investigated miRNAs in SCNT pregnancies. In contrast, several studies have focused on the use of circulating miRNAs as diagnostic biomarkers of reproductive problems^28^, early pregnancy^24^, gestational monitoring^22^ and placental functions^18,22,29^.

Regarding the use of miRNAs as a tool to investigate pregnancy outcome, Ioannidis and Dounadue (2016) observed an increase in abundance of some miRNAs on gestational days 16 to 24 in Holstein cattle. More specifically, they found that the bta-miR-26a level was correlated with early pregnancy in cattle. Additionally, they found 16 other miRNAs with different abundance levels between pregnant and non-pregnant cows. In humans, it has been demonstrated that increasing levels of hsa-miR-26a in the maternal circulation are associated with pregnancy. However, this miRNA has also been associated with a human placental disease (preeclampsia) and might be considered a marker of a poor prognosis for human gestations^19^. The miRNA bta-miR-26a was detected in all of our samples, with similar abundance levels observed among the groups. It is important to emphasize that in our experimental groups, all recipients were pregnant on the 21^st^ day of gestation when the blood samples were collected. Our data suggest that, in cattle, bta-miR-26a may be involved in early pregnancy and its diagnosis, but not in the quality and maintenance of pregnancy. These results corroborated the findings described above. Among the 16 miRNAs with different abundance levels in pregnant vs. non-pregnant cows reported by Ioannidis and Dounadue (2016), only seven (bta-let-7d, bta-let-7f, bta-miR-101, bta-miR-103, bta-miR-29a, bta-miR-29c and bta-miR374b) were found to have different abundance levels among the groups in our study. Of these seven miRNAs, all except bta-miR-374b showed low abundance levels in the C-EPL group compared with the C-LTP and AI-LTP groups, which showed similar abundance levels. Additionally, bta-miRNA-374b showed an abundance similar to that observed in the AI-LTP group. Furthermore, of the 40 miRNAs with different abundance levels among our groups, 27 exosomal-miRNAs (67.5%) were present at low levels in the plasma of recipient cows from the C-EPL group compared with the other two experimental groups (C-LTP and AI-LTP). A striking feature of our study was that we did not observe any miRNAs with high abundance levels in the group that experienced spontaneous abortion (C-EPL) compared with the other two groups in which the gestations reached term (C-LTP and AI-LTP). Additionally, the C-LTP and AI-LTP groups showed quite similar miRNA abundance levels. This observation demonstrated a notably low abundance level of exosomal-miRNAs in defective SCNT embryo pregnancies. At the moment, the mechanism underlying this low abundance is unclear.

In humans, high levels of some miRNAs (i.e., hsa-miR-29a and hsa-miR-103) appear to be associated with poor gestational prognosis. Low levels of hsa-miR-16 have also been associated with gestational problems^13,19^. In contrast with this finding, we detected different abundance patterns of bta-miR-29a and bta-miR-103 in our samples; these miRNAs were highly abundance levels in the groups that reached term (C-LTP and AI-LTP). Nevertheless, the abundance level of bta-miR-16 in the samples from cows that experienced spontaneous abortion (C-EPL) was similar to that observed in humans. These results suggested that in humans, either low or high abundance levels of circulating miRNAs can be associated with poor gestational outcome. However, based on our results, we did not observe the same relationship in cattle with SCNT pregnancies. Indeed, our observation was that the majority of miRNAs were present at a low level of abundance in the group that experienced spontaneous abortion (C-EPL), and no miRNAs were observed to have high abundance levels in this group compared with the other two groups (C-LTP and AI-LTP).

In a recent study, the abundance levels of 377 miRNAs in placentas at 50 days of gestation from embryos produced by SCNT, IVP or AI showed 278 miRNAs (73.7%) that were at a low level in the SCNT-derived placentas with two or more fold change and a P-value ≤ 0.05. Only five miRNAs (bta-miR-527, bta-miR-608, bta-miR-637, bta-miR-649 and bta-miR-938) were found with high levels in the SCNT placentas compared with the AI group^18^. Very low abundance levels of miRNAs have been reported in placentas of animals generated by assisted reproduction techniques (ARTs) *in vitro*, such as SCNT and IVF. These effects may be consequences of the abnormal epigenetic reprogramming that occurs as a result of these ARTs, since they are prone to failure. The aberrant reprogramming may affect chromatin structure, histone and DNA modifications and non-coding RNAs^18^. These altered epigenetic mark patterns cause errors in transcription that culminate in improper development during the earlier stages of SCNT embryo formation. In addition, there is strong evidence to suggest that SCNT fetuses possess malformed placentas. Since the placenta might contribute to exosomal-miRNA payloads, this may partially explain the notably low levels of miRNAs in placentas derived from SCNT or IVP^18^.

Generally, naturally conceived mammalian gestations have a low but expected incidence of embryonic and fetal losses. These losses mainly occur due to fetal abnormalities and ineffective maternal/fetal interactions. However, SCNT pregnancies normally show a high incidence of embryonic loss during the first trimester of pregnancy, and the placenta is implicated as a major contributor to pregnancy failure^30^. The C-EPL group, which contained cattle with SCNT-derived fetuses, showed abnormalities (detachment of membranes and undeveloped embryos) during pregnancy. On the other hand, gestations from the C-LTP and AI-LPT groups reached term without apparent problems. In our view, this demonstrates a high similarity between these two groups, and this homogeneity is reflected by their respective miRNA profiles.

It is worth noting that in our experiment, all exosomal-miRNAs found at different abundance levels were circulating in the maternal blood, and, therefore, we do not know the exact origins of these molecules. However, our findings suggested that the low levels of exosomal-miRNAs observed could be the result of poor nuclear reprogramming that is inherent to the SCNT procedure, since only samples from pregnancies that underwent early spontaneous abortion (C-EPL) showed low levels of these miRNAs. In addition, it is known that the placenta may contribute to the circulating miRNA profile in the maternal blood. In this way, the miRNAs with low levels in our samples could have originated from the placenta. Indeed, previous studies have reported that in placentas derived from embryos produced by SCNT, most miRNAs are with low levels^18^. Finally, our results showed that the low abundance levels of these exosomal-miRNAs in the maternal blood appear to be related to the poor gestational prognosis of SCNT animals, at least in cattle.

To gain insight into the pathways that might be deregulated in the animals described in this study, we completed a bioinformatics analysis of the 27 miRNAs with low abundance levels. This analysis revealed several relevant signaling pathways and functional annotation clusters related to the predicted target genes. In general, the signaling pathways identified here are involved in important cellular and biological processes, such as cancer, DNA repair and stability, cell proliferation and differentiation, angiogenesis, control of programed cell death or apoptosis, and cell survival.

Initially, when we analyzed the relationships among the target genes from the selected miRNAs, we identified many signaling pathways related to cancer, and we did not relate these pathways with the success of pregnancy or even with fetal development. However, the successful placentation is the only way to provide an adequate blood and nutrient supply for correct development and fetal growth. The placenta is a proliferative and vascularized structure with growth and differentiation behaviors similar to those described for tumors. These placental characteristics resemble the proliferation and invasion of cancer cell lines, and therefore, these cells share some similar mechanisms^31^. In this manner, miRNAs involved in cancer, which are defined as oncomiRs, can be analyzed during pregnancy and are potentially important during the placentation process^32^.

The Wnt, TGF-β and dorso-ventral axis formation pathways are critical to important biological events during the establishment and progression of pregnancy. Specially, the Wnt signaling pathway is conserved in metazoan animals and regulates cellular processes during embryo development, including cell fate determination, motility, polarity, primary axis formation and organogenesis^33^. In humans, deregulated Wnt signaling can bring catastrophic consequences for the developing embryo and is a causative factor for many pathologies, most notably some cancers, skeletal defects and birth defect disorders^34^. The TGF-β signaling pathway has a profound impact on reproductive function. In mammals, TGF-β ligands are involved in processes such as ovulation, fertilization, and pregnancy establishment and maintenance^35^.

Another relevant pathway identified in our study was the p53 signaling pathway, which is regarded as the guardian of the genome and regulates cellular response induced by various kinds of stress^36^. Depending on the stress signal received, the p53 pathway may inhibit angiogenesis and cell proliferation or activate programmed cell death and apoptosis. Additionally, previous studies have suggested that hypoxia may cause apoptosis in a trophoblast lineage and the p53 signaling pathway may be involved in this process^37^.

In humans, implantation failure is the most common problem associated with IVF and embryo transfer^37^. In SCNT animals, fetal mortality is also associated with inappropriate placental and embryo development^7,11,30,38,39^. As discussed above, the predicted target genes of the 27 miRNAs that were observed to have low abundance levels during early pregnancy loss in SCNT embryos are involved in the p53 signaling pathway. Additionally, this pathway is involved in the activation of apoptotic processes during early placental development. In this manner, both the poor placentation and fetal development demonstrated in the C-EPL group in relation to the C-LTP group might have affected pregnancy development and led to the miscarriage of these SCNT embryos. We investigated the poor placental vascularization in these pregnancies, but our analysis did not identify differences between the C-EPL and C-LTP groups at the 21^st^ day of gestation. When we analyzed the top 10 functional annotation clusters, the following three were highly relevant to our findings: i) angiogenesis (blood vessel development and vascular morphology); ii) embryonic morphogenesis and iii) in utero embryonic development. Thus, problems affecting these important biological processes during the early establishment of pregnancy can have critical consequences for correct gestational development.

In conclusion, our results demonstrated a notably low abundance level of exosomal-miRNAs in the maternal blood of cattle recipients that underwent spontaneous abortions during the early stages of SNCT-derived pregnancies. Furthermore, the top 10 signaling pathways and the top 10 functional annotation clusters of the predicted genes regulated by the 27 miRNAs with different levels among the groups were associated with the establishment of early pregnancy and embryonic development. In this way, these miRNAs could be used as biomarkers for the prognosis of early SCNT-derived pregnancies in cattle. Finally, it is tempting to speculate that specific failures in these signaling pathways are associated with the gestational problems identified in our analysis, including placental and embryonic development and abortions. However, we did not carry out experiments to interfere with these signaling pathways and support these findings. These findings warrant further investigation to validate the miRNAs and their target genes and to determine their definitive roles in pregnancy establishment and embryonic development in cloned cattle.

## Methods

### Ethics statement

This study was conducted in accordance with ethical guidelines of research and animal care. The protocol was approved by the Ethic Committee on Animal Use of the School of Animal Science and Food Engineering - University of Sao Paulo (CEUA/FZEA) - (Protocol Number: CEUA n° 2210140815).

### SCNT embryo production

The bovine SCNT embryos were produced as previously described with a few modifications^40^. Previously, a primary fetal fibroblast cell line was established *in vitro* and used in all SCNT-replicates. For the cloning process, bovine ovaries were collected from a slaughterhouse and transported to the laboratory in a saline solution (NaCl 0.9%) containing antibiotics (50 µg/mL gentamicin) at 37 °C. The oocytes were aspirated, selected and *in vitro* matured (IVM) in TCM-199 bicarbonate-buffered medium supplemented with 10% fetal bovine serum (FBS), 50 µg/mL hCG (Vetecor; Ourofino Saúde Animal, Cravinhos, Brazil), 0.5 µg/mL follicle stimulating hormone (Folltropin; Ourofino Saúde Animal), 0.2 mM pyruvate and 50 µg/mL gentamicin. After 19 h of IVM, the oocytes were denuded, and those at the metaphase II (MII) stage with evident 1^st^ PB were selected. MII oocytes were then incubated for 15 min in Synthetic Oviductal Fluid (SOF) medium supplemented with 7.5 µg/mL cytochalasin B and 1 µg/mL Hoescht 33342. After the incubation period, the oocytes were enucleated in PBS supplemented with 10% FBS, 2 mM sodium pyruvate and 50 µg/mL gentamicin. The 1^st^ PB and the metaphase plate were removed by aspiration of the adjacent cytoplasm, and the fibroblasts were transferred one-by-one into the perivitelline space of the enucleated oocytes. The resulting couplet was placed in a fusion chamber (Eppendorf) filled with electrofusion solution (0.28 M mannitol, 0.1 mM MgSO4, 0.5 mM HEPES, and 0.05% BSA in water) and subjected to a single pulse of a continuous current (1 KV/cm for 5 s) followed by two pulses of alternating current (1.75 KV/cm for 45 µs) to promote fusion between the fibroblast and the recipient ooplast. The artificial activation was carried out in H199 medium (TCM-199 with 25 mM Hepes) supplemented with 0.001 mg/mL BSA and 5 μM ionomycin for 5 min. The presumptive zygotes (PZs) were then washed in H199 medium saturated with 30 mg/mL BSA and incubated in SOF medium supplemented with 5 mg/mL BSA, 2.5% FCS, 0.2 mM sodium pyruvate, gentamicin, and 2 mM 6-DMAP for 3 h. The PZs were transferred to *in vitro* culture (IVC) in SOF medium for 7 days.

### SCNT embryo transfer into recipient cows

SCNT blastocysts were transferred individually on the 7^th^ day of culture to previously synchronized recipient cows^41^. These recipient cows were evaluated during the early stages of pregnancy for fetal heartbeat by ultrasound visualization on D28. Pregnancies were allowed to develop to term or until spontaneously aborted, and the recipient cows were then grouped according to pregnancy outcome. The pregnancies with SCNT embryos were classified in two different groups: (1) clone - early pregnancy loss (C-EPL): pregnancies that spontaneously aborted before the end of the first trimester of gestation and (2) clone - late pregnancy (C-LTP): pregnancies that reached term. Artificial inseminations were performed in parallel and monitored until development to term and designated the control group also known as the artificial insemination - late pregnancy (AI-LTP) group. The SCNT pregnancies were generated from a cell linage derived from a single male, and all pregnancies (clones and AI) were from confirmed male embryos, ruling out the possibility that the differences observed were related to sex.

### Pregnancy monitoring

All recipients were examined by transrectal ultrasonography in mode B and color Doppler using MyLab 70 Vet Gold (Esaote Healthcare, Italy) equipment with a transrectal multifrequency linear transducer for use in large animals (LV513 Esaote Healthcare, Italy). Ultrasonographic monitoring began on day 14 after ovulation and continued every two days until embryo vesicle detection. Afterwards, the monitoring continued every two weeks until the end of the first trimester. During examination, uterine horns were assessed individually to detect gestational fluids, placental membranes and the embryo or fetus. During the advanced stages of pregnancy, the cotyledons were also evaluated, and embryonic loss was confirmed by failure to detect a fetal heartbeat.

### Maternal blood sample collection and plasma preparation

Blood samples were collected from all recipients on D7 of the estral cycle, at the time of embryo transfer for the SCNT groups (C-EPL and C-LTP) or seven days after artificial insemination in the AI-LTP group. Additionally, we collected blood samples on the 21^st^ day of gestation. The samples were collected on D21, which was based on the timing of maternal recognition of gestation in cows (15 to 19 days after fertilization)^42^. Blood samples were collected in a Vacutainer™ tube with EDTA from the recipient’s caudal vein. Samples were centrifuged at 400 x g for 30 min to ensure complete separation of the plasma. In addition, three sets of centrifugation were performed to remove all intact blood cells or debris from the plasma. The first centrifugation was at 200 x g for 10 min, the second was at 1.500 x g for 15 min and the third was at 16,000 x g for 30 min. All centrifugations were performed at 4 °C. The plasma samples were then stored at −80 °C until processing for exosome isolation and RNA extraction.

### Exosome isolation and RNA extraction

Exosomes were isolated from 400 μL of plasma. For this purpose, 100 μL of Exoquick^TM^ (System Biosciences) was added to each sample, which was then mixed and incubated overnight at 4 °C. Extracellular vesicles were then precipitated by centrifugation at 1,500 x g for 30 min. The supernatant was discarded, and the extracellular vesicle pellet was carefully dissolved in 200 µL 1X Ca^+2^- and Mg^+2^-free PBS. The isolated exosomes were characterized by transmission electron microscopy, western blot and morphological examination and quantified by Nanoparticle Tracking Analysis (Supplementary Figure 3). Total RNA extraction was performed by the addition of 750 μL TRIZOL LS (TRI BD, Sigma Aldrich, St. Louis, MO) and 8 μL polyacryl to the extracellular vesicle pellet according to the manufacturer’s instructions. Total RNA was eluted in RNAse/DNAse-free water. To remove possible genomic DNA contamination, samples were incubated at room temperature for 15 min in the presence of 1 µL DNAse and 1 µL DNAse buffer (Life Technologies, Carlsbad, CA). After incubation, the reaction was stopped by the addition of 1 µL inactivation reagent, and the samples were heated to 65 °C for 10 min. The RNA concentration was determined using a NanoDrop 1000 spectrophotometer (NanoDrop Technologies). RNA aliquots were stored at −80 °C until further use.

### cDNA synthesis and quantitative PCR analysis

Reverse transcription (RT) was performed using the Reverse Transcriptase Kit II miScript (Qiagen, Valencia, CA) according to the manufacturer’s recommendations. An initial 200 ng RNA was used with 2 uL 5x miScript Hiflex, 1 µL 10x miScript nucleic acid mix, 1 µL miScript enzyme (RT) and RNAse/DNAse-free water when necessary to achieve a total reaction volume of 10 µL. The samples were incubated at 37 °C for 1 h and at 95 °C for 5 min to inactivate the miScript enzyme (RT). Next, a customized panel of miRNA primers containing in a total of 348 specific-bovine miRNAs and three miRNAs consistently detected among the groups (miR-99b, RNU1, and RNU43) was used to evaluate the abundance levels of these miRNAs^43–45^. The primer sequences were obtained from the miRBase database (www.mirbase.org) and designed utilizing the mature sequence as the forward primers. For the qPCR reaction, cDNA was diluted by adding 40 µL water (1:5 dilution) to the final reaction volume. The mix was prepared with 500 μL SYBR Green (Qiagen), 290 μL water, 100 μL 10x Universal Primer miScript and 10 μL cDNA. The qPCR was performed in 96-well plates, and the final reaction volume was 10 μL. In each well, 9 μL mix and 1 μl forward primer were added. The master mix and primers were pipetted into the wells of each plate with the assistance of a robotic pipetter (Hamilton Microlab STARlet) according to the manufacturer’s recommendations. The qPCR cycling conditions consisted of an initial incubation at 95 °C for 15 min and 40 cycles at 95 °C for 15 sec, 55 °C for 30 sec, and 72 °C for 30 sec. The melting-curve analysis was used to confirm the amplification of a single PCR product. A miRNA was considered detected if the amplification occurred at a crossing-point value (Ct) less than 37 cycles.

### Pathway and functional annotation cluster analysis of miRNAs using bioinformatics

MiRNAs with different abundance levels among the three groups were analyzed individually using the website TARGETSCANHUMAN - Prediction of miRNA targets (TARGETSCANHUMAN release 7.0 - http://www.targetscan.org/vert_70/) to predict the bovine genes regulated by each miRNA. The top 80 genes were identified (code Ensembl Transcript ID) and selected for each miRNA. Subsequently, all of these selected genes were simultaneously analyzed using the website DAVID - Functional Annotation Tool (DAVID Bioinformatics Resources 6.7, NIAID/NIH - https://david.ncifcrf.gov/summary.jsp). Finally, the signaling pathways and functional annotation clusters were arranged by fold change and enrichment score, respectively. The top 10 signaling pathways and the top 10 functional annotation clusters involved with all miRNAs were presented and discussed herein.

### Statistical analysis

The miRNA abundance levels were measured in three replicates (biological replicates) for the C-EPL and C-LTP groups and four replicates for the AI-LTP (control) group. The data were normalized (ΔCt) according to the geometric mean of the three miRNAs consistently detected among the groups. Normalized data were transformed into 2^−ΔCt^ to calculate the abundance level. The normalized values were subjected to analysis of variance (ANOVA), and the means were compared by Tukey’s test using the general linear model procedure (GLM) in SAS (Statistical Analysis System, version 9.3) at a significance level of 5%. The p-values of miRNAs were adjustment for multiple testing and all the miRNAs that passed on this test were considered significant among the groups. For heatmap and dendrogram analysis and generation as well as principal component analysis (PCA), the normalized values (2^−ΔCt^) were exported and processed with the assistance of Metaboanalyst 3.0 (www.metaboanalyst.ca <http://www.metaboanalyst.ca/>)^46^.

## Electronic supplementary material

Supplementary Dataset 1

Supplementary Dataset 2

Supplementary Dataset 3
