# Supplementary material for: Low levels of exosomal-miRNAs in maternal blood are associated with early pregnancy loss in cloned cattle
Source: Sci Rep. 2017 Oct 30;7:14319. doi: 10.1038/s41598-017-14616-1 (PMC5662615; doi:10.1038/s41598-017-14616-1)
Supplement: Supplementary file 1 — Supplementary Dataset 1 [file 41598_2017_14616_MOESM1_ESM.doc]

**Article Title:** Low levels of exosomal-miRNAs in maternal blood are associated with early pregnancy loss in cloned cattle.

**Authors:** De Bem THC*, da Silveira JC, Sampaio RV, Sangalli JR, Oliveira ML, Ferreira RM, Silva LA, Perecin F, King WA, Meirelles FV, Ramos, ES.

**Supplementary File - Figures**

**Supplementary Figure 1**. Heatmap depicting the average Ct values from the 27 miRNAs with different abundance levels in the maternal blood at the 21^st^ day of gestation among the following three groups evaluated: Clone - Early Pregnancy Loss (C-EPL), Clone - Late Pregnancy (C-LTP), Artificial Insemination - Late Pregnancy (AI-LTP). The higher miRNA levels are shown in red, whereas the lower miRNA levels are in blue.


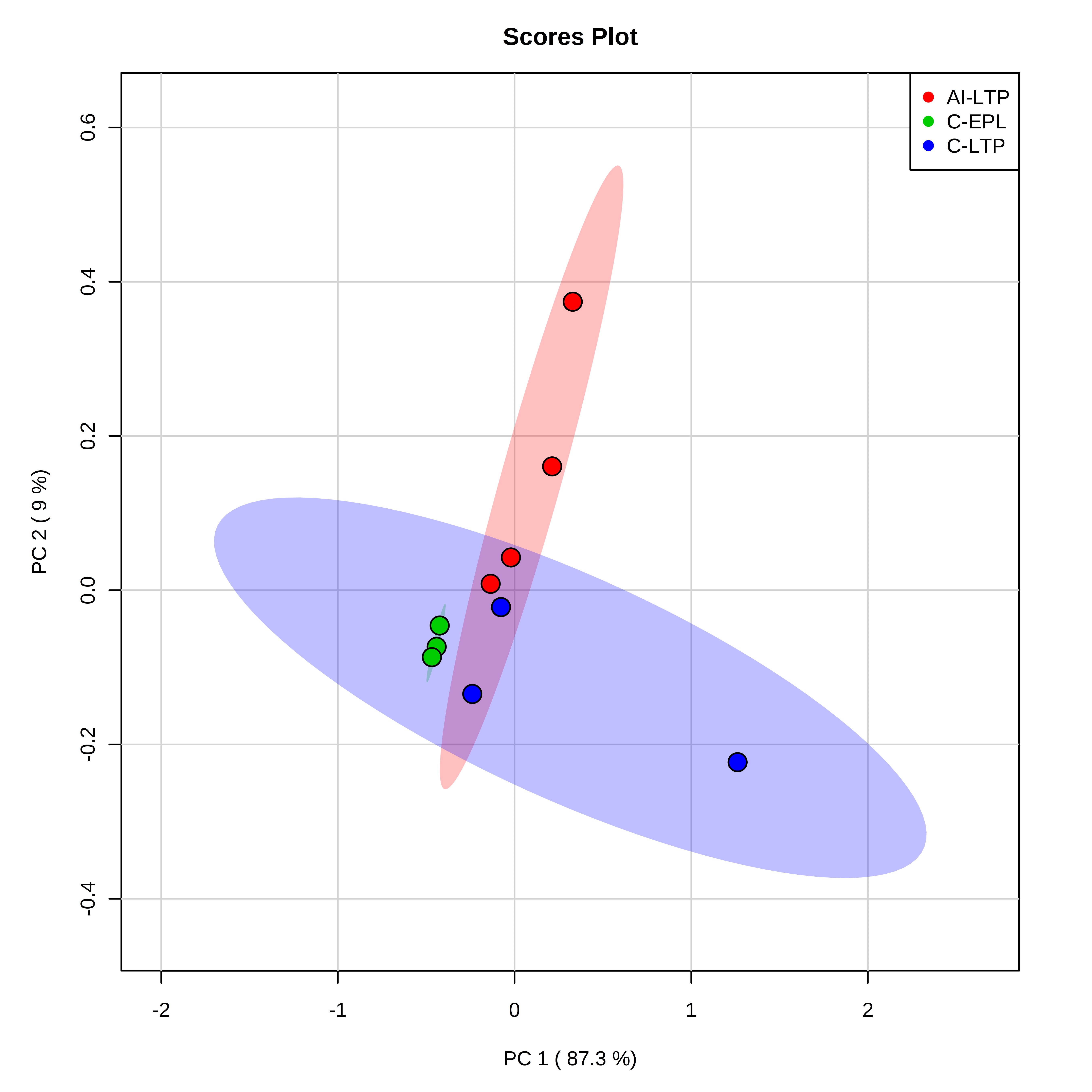


**Supplementary Figure 2.** Principal component analysis (PCA) plot using the data from the 40 exosomal-miRNAs with different abundance levels on the 21^st^ day of gestation among the three groups analyzed by qRT-PCR. C-EPL: green dots; C-LTP: blue dots; and AI-LTP: red dots.

**Supplementary Figure 3**. Characterization of small-extracellular vesicles after isolation using ExoQuick^TM^. **A)** Transmission electron microscopy of isolated small-extracellular vesicles (Scale bar 100nm). **B)** Western blot of proteins in small-extracellular vesicles derived from maternal plasma of the three analyzed groups (C-EPL, C-LTP and AI-LTP). Herein we identified the presence of ALIX and CD63 two extracellular vesicles markers, present in all samples. Additionally, we verified the presence of Calnexin, a reticulum endoplasmic protein and Tomm20, a membrane mitochondrial protein, which serves as a negative control for cell contamination. **C)** Morphological characterization and quantification of small-extracellular vesicles by Nanoparticle Tracking Analysis. Nanoparticle Tracking Analysis demonstrated calculated size distribution is depicted as a mean (black line) with standard error (red shaded area). The mode size and total particle number are shown for each analyzed group.
